# Editorial: Enhancing adrenal tumor diagnostics: biomarkers and molecular mechanisms

**DOI:** 10.3389/fendo.2026.1843915

**Published:** 2026-04-15

**Authors:** Piotr Glinicki, Henrik Falhammar

**Affiliations:** 1 EndoLab Laboratory, Centre of Postgraduate Medical Education, Warsaw, Poland; 2 Department of Endocrinology, Centre of Postgraduate Medical Education, Warsaw, Poland; 3 Department of Endocrinology, Karolinska University Hospital, Stockholm, Sweden; 4 Department of Molecular Medicine and Surgery, Karolinska Institutet, Stockholm, Sweden

**Keywords:** adrenal mass, adrenocortical carcinoma, biomarkers, Cushing syndrome, paraganglioma, pheochromocytoma, promary aldosteronism

The diagnosis and classification of adrenal tumors remain a major challenge that involves various fields of medicine (1). New opportunities in biochemical and molecular testing involve various omics-based techniques that enable the comprehensive analysis of numerous analytes, compounds, proteins, peptides, and nucleic acids in various biological matrices; this comprehensive analysis allows for an assessment of the utility of these tests throughout the entire diagnostic and therapeutic process (2–5). Moreover, new imaging techniques allow for a more detailed assessment of pathological changes observed in the diagnosis of adrenal tumors.

The current Research Topic remains of high scientific and clinical interest and includes 12 articles.

Bernardi et al. described a novel and previously not reported germline variant in the *SDHB* gene in a patient with a metastatic sympathetic paraganglioma (PGL) presented with hypertension, tachycardia and hyperhidros. Debulking surgery as well as genetic testing were scheduled. The authors concluded that the clinical features and immunohistochemical findings regarding the *SDHB* gene provided a solid basis for classifying the new germline *SDHB* c.314T>A genetic variant as a class 4 variant, i.e., “likely pathogenic”.

Pauzi et al. presented an article titled “*Histopathological spectrum of common aldosterone-driver 40 gene mutant aldosterone-producing adenomas (APAs)*,” where the authors analyzed 33 APAs with 41 confirmed aldosterone-driver variants (17 KCNJ5 variant APAs, 8 ATP1A1 variant APAs, 6 42 CACNA1D variant APAs, and 2 CTNNB1 variant APAs). The adenomas were immunohistochemically stained using43 H&E, CYP17A1, CYP11B2, KCNJ5, Ki67, β-catenin, and LHCGR antibodies.” Results of this study support the suggestion that *CYP11B2*-guided sequencing of all the currently known aldosterone-driver genes can fine tune current genotype-phenotype histopathology profiles.

The review article by Szatko et al. described the current challenges of biochemical diagnostics (immunoassays) of primary aldosteronism, including screening, case confirmation and subtyping and novel “omics” techniques in the diagnostics (steroidomics and proteomics).

Adrenocortical carcinoma (ACC) presenting as pheochromocytoma is rare in the literature. In a case report and mini-review [Sobolewska et al.] authors reported on ACC cases clinically manifesting as pheochromocytomas, including a novel clinical case of a 59-year-old female.

Peng et al, presented the diagnostic value of ^18^F-AlF-NOTA-Pentixafor PET/CT in subtyping primary aldosteronism in 88 patients. The CXCR4-targeted ^18^F-AlF-NOTA-pentixafor PET/CT was a valuable noninvasive tool for diagnosing unilateral primary aldosteronism, demonstrating high sensitivity and specificity.

Mytareli et al. presented an original research to validate the diagnostic performance of selected circulating microRNAs (miR-483-5p, miR-210, miR-335 and miR-22-3p), identified through microRNA profiling studies, as markers of malignancy or cortisol hypersecretion in a cohort of patients with adrenal tumors. This study supports the potential of a liquid biopsy approach as an innovative method for diagnosing and monitoring patients with adrenal tumors, offering a complementary tool to existing diagnostic methods.

In a case report and literature review by Woźniak et al. entitled “*A 17-hydroxyprogesterone-secreting adrenal adenoma mimicking nonclassical 21-hydroxylase deficiency*” highlighted the occurrence of rare causes of benign adrenal adenomas with steroidogenesis disorders, which may lead to a misdiagnosis of congenital adrenal hyperplasia (CAH).

Weng et al. explored the value of serum steroid profiling as a complementary biomarker for malignancy diagnosis of ACC other than diameter, and explored the influence of sex and functional status. In this retrospective study, a matched cohort of patients diagnosed with either ACC or adrenocortical adenomas (ACA) based on histopathology was meticulously paired in a 1:1 ratio according to sex, age and functional status. Serum steroid profiling served as a helpful discriminative marker for ACC and ACA, with 11-deoxycortisol being the most valuable one. For other steroid hormones, consideration of sex differences and functional status was crucial.

Apsan et al. investigated the relationship between adiponectin, leptin and visfatin concentrations to metabolic risk factors and androgen concentrations in children and young adults with CAH. The conclusion was an inverse relationship between adiponectin and androstenedione which suggests that better CAH control can reduce the risk of insulin resistance and metabolic syndrome. However, a high glucocorticoid dose appeared to increase the risk of insulin resistance, underscoring the delicate balance required when treating CAH.

Zheng et al. described a novel *PRKAR1A* gene variant in a Chinese patient with Carney complex and reviewed the literature to enhance the understanding of Carney complex.

A mini-review by Olejarz et al. discussed publications from the past two years on changes in lipoprotein concentrations in endocrine disorders and provided an overview of the latest findings in this rapidly evolving and extensive field.
